# Short-Chain Dehydrogenase NcmD Is Responsible for the C-10 Oxidation of Nocamycin F in Nocamycin Biosynthesis

**DOI:** 10.3389/fmicb.2020.610827

**Published:** 2020-12-17

**Authors:** Xuhua Mo, Hui Zhang, Fengyu Du, Song Yang

**Affiliations:** ^1^Shandong Province Key Laboratory of Applied Mycology, School of Life Sciences, Qingdao Agricultural University, Qingdao, China; ^2^School of Chemistry and Pharmacy, Qingdao Agricultural University, Qingdao, China

**Keywords:** nocamycin, gene inactivation, biosynthetic pathway, Saccharothrix syringae, short chain dehydrogenase/reductase

## Abstract

Nocamycins I and II, featured with a tetramic acid scaffold, were isolated from the broth of *Saccharothrix syringae* NRRL B-16468. The biosynthesis of nocamycin I require an intermediate bearing a hydroxyl group at the C-10 position. A short chain dehydrogenase/reductase NcmD was proposed to catalyze the conversion of the hydroxyl group to ketone at the C-10 position. By using the λ-RED recombination technology, we generated the *NcmD* deletion mutant strain *S. syringae* MoS-1005, which produced a new intermediate nocamycin F with a hydroxyl group at C-10 position. We then overexpressed *NcmD* in *Escherichia coli* BL21 (DE3), purified the His_6_-tagged protein NcmD to homogeneity and conducted *in vitro* enzymatic assays. NcmD showed preference to the cofactor NAD^+^, and it effectively catalyzed the conversion from nocamyin F to nocamycin G, harboring a ketone group at C-10 position. However, NcmD showed no catalytic activity toward nocamyin II. NcmD achieved maximum catalytic activity at 45°C and pH 8.5. The kinetics of NcmD toward nocamycin F was investigated at 45°C, pH 8.5 in the presence of 2 mM NAD^+^. The *K*_m_ and *k*_cat_ values were 131 ± 13 μM and 65 ± 5 min^−1^, respectively. In this study, we have characterized NcmD as a dehydrogenase, which is involved in forming the ketone group at the C-10 position of nocamycin F. The results provide new insights to the nocamycin biosynthetic pathway.

## Introduction

The short-chain dehydrogenases/reductases (SDRs), one of the largest protein families, distribute in all kinds of organisms. Despite low residue identities in pairwise comparisons, all SDRs share a Rossmann fold-type domain for NAD(P)^+^ binding (Kavanagh et al., 2008; Persson and Kallberg, 2013). SDRs have been classified into seven families: classical, extended, atypical, intermediate, divergent, complex and unassigned, and the classical type is the most prominent (Persson and Kallberg, 2013; Gräff et al., 2019). SDRs show diverse substrate spectra, including steroids, alcohols, sugars, aromatic compounds, and xenobiotics, and for this reason, more and more SDRs have been extensively explored for industrial production (Persson and Kallberg, 2013; Luo et al., 2019; Savino et al., 2019; Shanati et al., 2019; Zhou et al., 2019; Su et al., 2020). SDRs play diverse roles in core metabolism and specific metabolism pathways such as steroidal metabolism, detoxification and drug resistance (Sonawane et al., 2018; Laskar and Younus, 2019). Moreover, SDRs play important roles in biosynthetic pathways of microbial secondary metabolites (MacKenzie et al., 2007; Mattheus et al., 2010; Bown et al., 2016).

**Figure 1:**
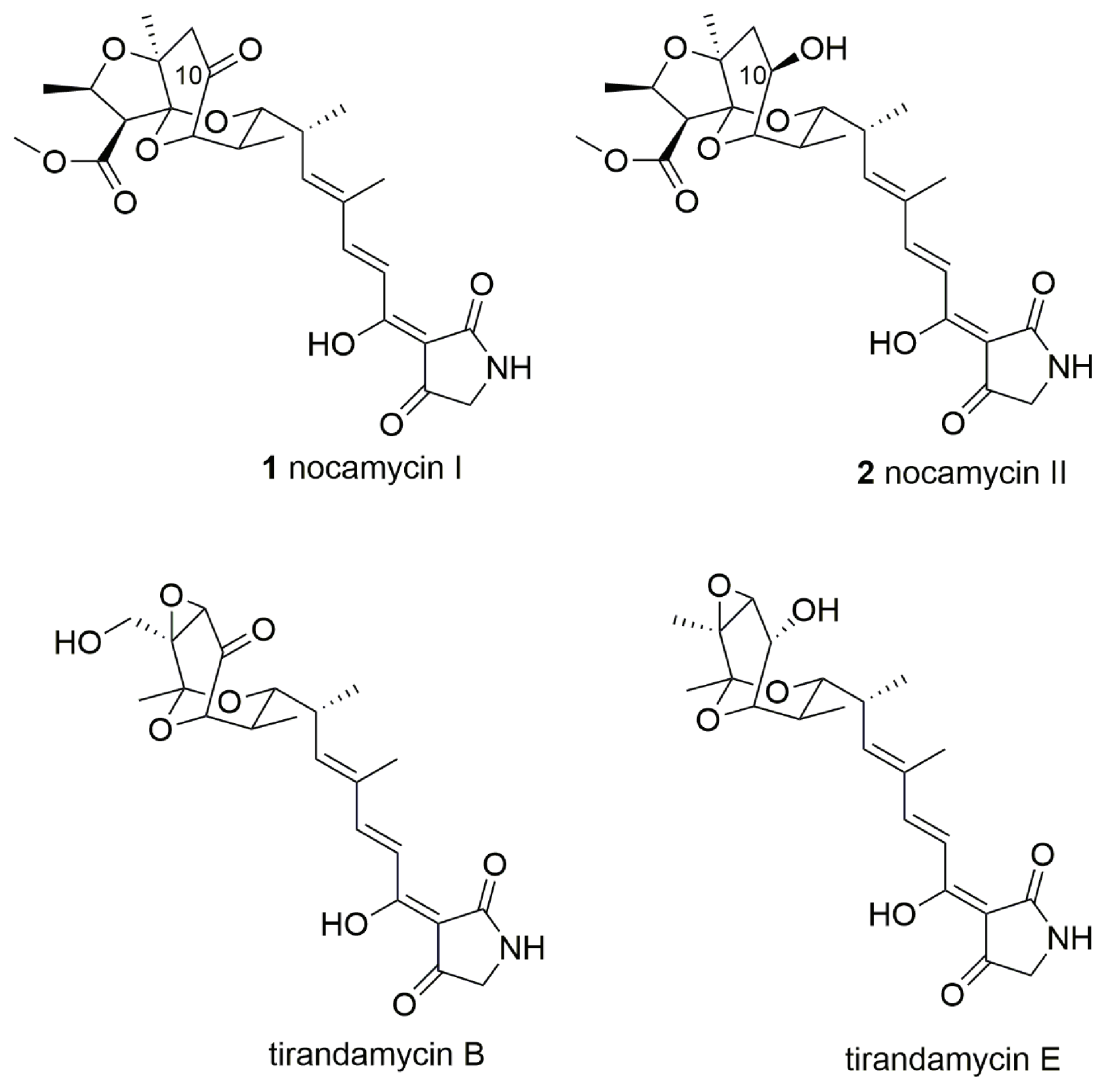
The nocamycin I, nocamyicn II, and related structures.

Nocamycins I and II, isolated from the broth of *Saccharothrix syringae* NRRL B-16468, feature two unique structural moieties, namely tetramic acid (2,4-pyrrolidinedione) and bicyclic ketal scaffolds (Figure 1; Gauze et al., 1977). Nocamycin I displays potent and broad antimicrobial activities against a panel of Gram-positive and Gram-negative bacteria, especially toward some anaerobic bacteria such as *Bacteroides fragilis*, *Clostridium* sp., *Fusobacterium* sp., and *Sphaerophorus* sp. with minimum inhibitory concentrations (MICs) in the range of 0.1–0.4 μg/ml (Tsukiura et al., 1980; Tsunakawa et al., 1980; Bansal et al., 1982). In addition, the carboxylate O-methyl group appears to be essential for nocamycins’ antibacterial property. Nocamycin E, lacking the carboxylate O-methyl group, shows less antibacterial activity (Mo et al., 2017a).

The gene cluster responsible for nocamycins biosynthesis has been identified from *S. syringae* NRRL B-16468 in 2017 (Mo et al., 2017b). The skeleton of nocamycins is assembled by a hybrid type I polyketide synthases (PKS) and non-ribosomal peptide synthetase (NRPS) system (Mo et al., 2017b). The gene cluster for nocamycins consists of 21 open reading frames (ORFs), which includes five genes encoding for type I PKS, one gene encoding for NRPS, one gene encoding for a Dieckmann cyclase NcmC, one gene encoding for a SDR NcmD, two genes encoding for cytochrome P450 oxidases NcmO and NcmG, one gene encoding for a glycoside dehydratase NcmE, one gene encoding for a SAM dependent methyltransferase NcmP, and five genes encoding for regulatory proteins (Mo et al., 2017b). Among the 21 ORFs, the functions of several genes have been investigated. The results of gene disruption experiment indicated that the cytochrome P450 oxidase NcmG was involved in formation of furan ring (Mo et al., 2017b). Furthermore, gene disruption and biochemical assays clearly demonstrated that the SAM-dependent methyltransferase NcmP was responsible for formation of the carboxylic methyl ester (Mo et al., 2017a).

The disparity between nocamycins I and II is the ketone or hydroxyl group at C-10 position, and the similar structure has been observed in tirandamycin B and its biosynthetic intermediate tirandamycin E (Figure 1). For tirandamycins, a FAD dependent dehydrogenase TrdL/TamL has been shown to catalyze the formation of ketone group at C-10 position (Carlson et al., 2011; Mo et al., 2011). However, for nocamycins, the enzyme for formation of ketone group at C-10 position remains unclear. We initially inactivated the gene *ncmL* encoding for a FAD-dependent protein, and resultant research demonstrated that it exerted no impact on production of nocamycins (Mo et al., 2017b). By carefully examining the biosynthetic gene cluster, the SDR NcmD is proposed to be the candidate to catalyze the conversion from hydroxyl to ketone at C-10 position. In the current study, we have established that NcmD acts as a dehydrogenase and catalyzes the formation of C-10 ketone group in nocamycin biosynthetic pathway by using *in vivo* gene disruption and *in vitro* biochemical assays.

## Materials and Methods

### Bacteria, Medium, and Culture Conditions

*Saccharothrix syringae* NRRL B-16468 was used as producer of nocamycins (Mo et al., 2017b). *Escherichia coli* DH5α was used as host for general clone. *Escherichia coli* BL21(DE3) was used as host for protein expression. *Saccharothrix syringae* NRRL B-16468 and its derivative strain were maintained on ISP4 agar medium. The medium contained 1% soybean flour, 3% glycerol, 0.2% NaCl, and 0.2% CaCO_3_, pH 7.0 was used for fermentation of *S. syringae* and its derivative strain. All cultures for *S. syringae* were incubated at 28°C. The *E. coli* strains were cultured by using Luria-Bertani (LB) agar or liquid medium with appropriate antibiotics.

### Generating Mutant Strain *Saccharothrix syringae* pMoS-1005 (*ΔNcmD*)

The gene *NcmD* was inactivated by using λ-RED recombination technology according to the literature previously reported (Mo et al., 2017b). The primers NcmD-delF (5'-CTCGCCGAGGCGTTCGCGGCCGAGGGCGCCCGAGTGGTGATTCCGGGGATCCGTCGACC-3') and NcmD-delR (5'-CCGGTTCTCCCGCACGGCGTCGAGGGTCGCCGCGGCCACTGTAGGCTGGAGCTGCTTC-3') were used to amplify the fragment *oriT/acc(3)IV* cassette from the plasmid pIJ773, the resultant PCR fragment was used to replace partial gene region of *NcmD* in cosmid p5-C-9 to generate plasmid pMoS-1005. Then, the correct mutated plasmid pMoS-1005 was introduced into *E. coli* ET12567/pUZ8002, which then conjugated with wild type *S. syringae* spores as described previously (Mo et al., 2017b). The exconjugants were firstly selected by the phenotype of kanamycin sensitive (Kan^S^) and apramycin resistant (Apr^R^), and then their genotypes were further verified by using PCR with the primers NcmD-tF (5'-ATGCGCGAGTTGACCGACC-3') and NcmD-tR (5'-AGCACGTCCAGGAAGTCAC-3'). The desired double cross-over mutant strain was termed as *S. syringae* MoS-1005.

### Fermentation and Analysis of Mutant Strain *Saccharothrix syringae* MoS-1005

*S. syringae* wild type and mutant strain *S. syringae* MoS-1005 were cultured by using the method described previously (Mo et al., 2017b). After 7 days culture, the broth was extracted by ethyl acetate, subsequently, the organic phase was collected and evaporated into dryness, re-dissolved in methanol, and subject to high performance liquid chromatography (HPLC) analysis. Analytical HPLC was performed on Waters 2699 HPLC system (Waters Technologies Inc., United States) equipped with a PDA detector and a Welch Ultimate AQ-C18 ODS column (250 × 4.60 mm, 5 μm). The mobile phase contained solvent A and B. Solvent A consisted of 15% CH_3_CN in water supplemented with 0.1% formic acid. Solvent B consisted of 85% CH_3_CN in water supplemented with 0.1% formic acid. Samples were eluted with a linear gradient from 5 to 90% solvent B in 20 min, followed by 90 to 100% solvent B for 5 min, then 100% solvent B for 3 min, at a flow rate of 1 ml/min under UV detection at 355 nm.

### Isolation of New Nocamycin Derivative Produced by *Saccharothrix syringae* MoS-1005

For fermentation of *S. syringae* MoS-1005 in a large scale, 8 L liquid media were used by using a two-step fermentation procedure as described previously (Mo et al., 2017b). After incubation, the culture broths were collected and centrifuged. The supernatant broth was extracted by ethyl acetate for three times and the mycelia were extracted by methanol for three times. Then, the entire organic solvents were evaporated into dryness to yield crude extracts. The crude extracts were dissolved in a mixture of CH_2_Cl_2_:CH_3_OH (1:1), and then mixed with appropriate amount of silica gel (100–200 mesh, Qingdao Marine Chemical Corporation, China). The samples were applied on normal phase silica gel chromatography column and eluted with CH_2_Cl_2_:CH_3_OH (100:0–50:50) to give 10 fractions, and all of them were analyzed by HPLC. Fractions 4 and 5 containing the target compound were used for further purification on reverse phase C-18 silica gel (YMC, Japan) by using medium-pressure liquid chromatography (MPLC, Agela corporation, China). The fractions containing the target compound were combined and further purified by Sephadex LH-20 (GE healthcare, Sweden) gel filtration chromatography to afford the purified nocamycin F.

### Heterologous Production and Purification of Recombinant Protein NcmD

The *NcmD* gene was amplified from cosmid p5-C-9 by using PCR with primers NcmD-expF (5'-TAATAATCATATGCGCGAGTTGACCGACCG-3', underline is *Nde*I site) and NcmD-expR (5'-ATATGGATCCCTAGTCGGTCGGCGCGGCG-3', underline is *Bam*HI site). The resultant PCR products were digested by *Nde*I and *Bam*HI, and then inserted into pET-28a (+) vector digested with the same restriction enzymes to yield plasmid pMosD. After verification of the inserted gene fragment by sequencing, the plasmid pMosD was introduced into *E. coli* BL21(DE3) for protein expression.

*Escherichia coli* BL21 (DE3) strain carrying plasmid pMosD was grown in LB medium with 50 μg/ml kanamycin at 37°C to an OD_600_ = 0.6. Then, isopropyl-β-D-thiogalactopyranoside (IPTG) was added into the culture at a final concentration of 0.1 mM to induce the expression of NcmD for 18 h at 20°C. Subsequently, the cells were collected, centrifuged, and re-suspended in binding buffer (50 mM Tris-Cl buffer, 500 mM NaCl, and 10 mM imidazole, pH 7.9), and sonicated on ice. Cellular debris was removed by centrifugation (12,000 rpm, 30 min, 4°C). The supernatant was further purified by nickel-nitrilotriacetic acid (Ni-NTA) affinity chromatography according to the manufacturer’s protocol (Novagen, CA, United States). The purified protein was desalted by PD-10 column (GE Healthcare, United States) according to the manufacturer’s instructions. The purified protein NcmD was finally stored in 50 mM Tris-Cl buffer (pH 8.0) with 10% glycerol at −80°C for further enzymatic assays.

### *In vitro* Enzymatic Assays of NcmD With Nocamycin F and Nocamycin II

The enzymatic assays were firstly conducted at 30°C in 50 mM Tris-Cl buffer (pH 8.0) containing 200 μM nocamycin F, 2 μM NcmD enzyme, 2 mM NAD^+^ or NADP^+^ for an hour. Then, the catalytic activity of NcmD toward nocamycin II was performed in 50 mM Tris-Cl buffer (pH 8.0) containing 200 μM nocamycin II, 2 μM NcmD enzyme, 2 mM NAD^+^ at 30°C for an hour. The reaction mixtures without NAD^+^ or NADP^+^ were set as negative controls. To investigate the optimum temperature for NcmD, the assays were carried out in 50 mM Tris-Cl buffer (pH 8.0) containing 200 μM nocamycin F, 0.8 μM NcmD, 2 mM NAD^+^ at various temperature (T = 25, 30, 37, 40, 45, 50, 55, and 60°C) for an hour. For probing the effect of pH on NcmD, the 50 μl reaction mixtures containing 200 μM nocamycin F, 0.8 μM NcmD, 2 mM NAD^+^ were performed at 45°C at different pH buffer for an hour, including Tris-Cl buffer (50 mM, pH = 6.0, 6.5, 7.0, 7.5, 8.0, 8.5, and 9.0), NaHCO_3_-NaOH buffer (50 mM, pH = 10.0, 11.0). After quenching the reactions by adding 100 μl cold methanol, then, the samples were centrifuged, and the supernatants (30 μl) were subjected to HPLC analysis. For each reaction, three parallels were used.

To determine the kinetic parameters of NcmD toward nocamycin F, 100 μl reaction mixtures containing 0.2 μM NcmD, 2 mM NAD^+^ and varying nocamycin F (10 20, 40, 100, 150, 200, 400, and 600 μM) at pH 8.5, 45°C were conducted. After a pre-incubation at 45°C for 3 min, the reactions were initiated by adding substrate nocamycin F. At 2, 4, and 6-min, 30 μl reaction mixtures were taken, and then 60 μl ice-cold methanol was added and rigorously mixed by vortex. After centrifuge, 30 μl liquids were used for further HPLC analysis and quantified by a standard curve. The reaction rates were calculated and confirmed to be linear. The kinetics data were fitted to the Michaelis-Menten equation using Origin8.0 software. For each concentration of substrate, three replicates were conducted.

For elucidating the structure of the products obtained from the reactions with nocamycin F, 5 ml reaction mixtures containing 2 μM NcmD, 2 mM NAD^+^ and 6 mg nocamycin F were performed at pH 8.5, 40°C for 8 h. Then, the reaction mixtures were extracted by 10 ml ethyl acetate for three times. The target product was purified by preparative HPLC for NMR and high-resolution mass spectral (HR-MS) analysis.

### Spectroscopy Analysis of New Produced Nocamycin Derivatives

^1^H and ^13^C NMR spectra were recorded at 25°C on Bruker AV 500 instruments. LC-HR-MS data were acquired on a Waters micro MS Q-Tof spectrometer or a Thermo MAT95XP high resolution mass spectrometer.

## Results

### Bioinformatics Analyses of NcmD

**Figure 2:**
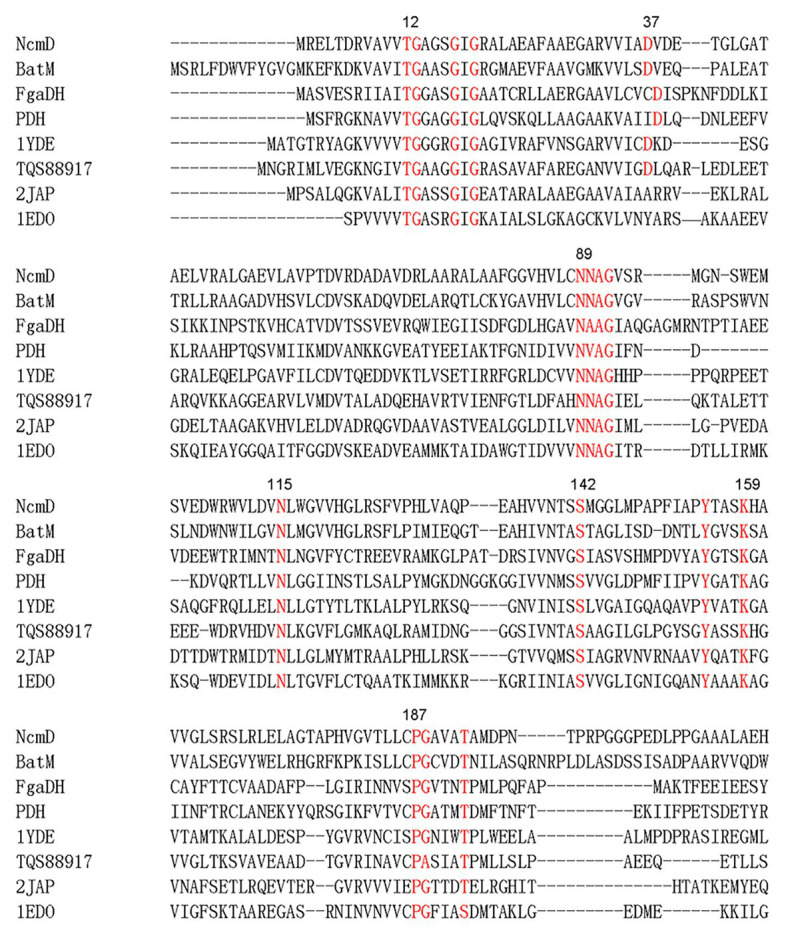
Amino acid sequences alignment analysis of NcmD and its closely homologous proteins and characterized short-chain dehydrogenases/reductases (SDRs). BatM originated from *Pseudomonas fluorescens* BCCM_ID9359 shows 40% identity to NcmD. 2JAP (CAD) involved in clavulanic acid biosynthetic pathway originated from *Streptomyces clavuligerus* shows 31% identity to NcmD. FgaDH originated from *Aspergillus fumigatus* is a NAD^+^ binding SDR. PDH originated from *Drosophila melanogaster* is a NAD^+^ binding SDR. The ephedrine dehydrogenase TQS88917 originated from *Arthrobacter* sp. TS-15 is a NAD^+^ binding SDR. 1YDE is a human retinal SDR using NADH as cofactor. 1EDO is a β-keto acyl carrier protein reductase from *Brassica napus* by using NADP^+^ as cofactor.

Within nocamycin biosynthetic gene cluster, the gene *NcmD* encoding for the SDR located next to the NRPS gene *NcmB*. NcmD shows identity to a series of SDRs, including 40% identity to BatM originated from kalimantacin/batumin-related polyketide antibiotic biosynthetic pathway (Mattheus et al., 2010), and 31% identity to clavulanic acid dehydrogenase (CAD) involved in clavulanic acid biosynthetic pathway (MacKenzie et al., 2007). Bioinformatics analyses revealed that NcmD shared the conserved motifs of classical SDR, namely, Rossmann fold NAD(P)-binding motif T_12_G_13_(X)_3_G_17_XG_19_, conserved catalytic triad S_142_(X)_12_Y_155_(X)_3_K_159_, N_89_NAG_92_ motif and P_187_G (X)_3_T_192_ motif (Figure 2; Filling et al., 2002; Persson and Kallberg, 2013; Gräff et al., 2019). In addition, an Asn_115_ residue (N) frequently served as an additional active site residue to make a catalytic triad is also conserved in NcmD (Figure 2). These bioinformatics analyses have demonstrated that NcmD is a classical SDR. As shown in Figure 2, an acidic residue aspartate (Asp, D) other than a basic residue occupies the position 37, indicating that the cofactor for NcmD is likely to be NAD^+^, which has been confirmed by other NAD^+^-preference SDRs such as FgaDH, PDH, 1YDE, and TQS88917 (Lukacik et al., 2007; Wallwey et al., 2010; Hofmann et al., 2016; Shanati and Ansorge-Schumacher, 2020).

### Construction, Analysis of *NcmD* Mutant Strain *Saccharothrix syringae* MoS-1005 and Isolation of the New Nocamycin Analog

**Figure 3:**
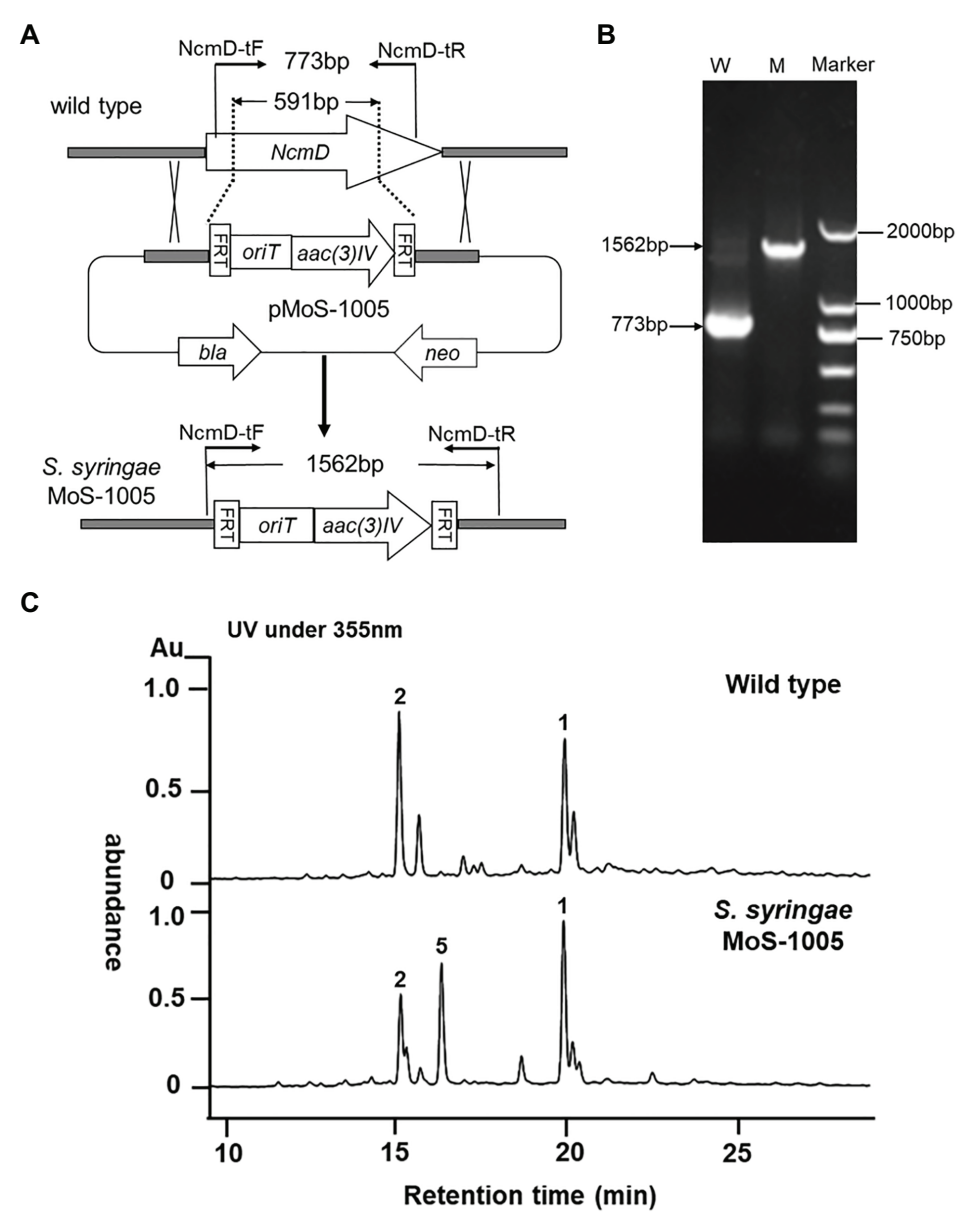
Generation of *ΔNcmD* mutant strain *Saccharothrix syringae* MoS-1005 and its metabolites profile analyzed by HPLC. **(A)** Schematic diagram of *NcmD* inactivation. The 591 bp internal *NcmD* fragment was replaced by *oriT* and *acc3(IV)* fragment in pMoS-1005. **(B)** PCR analysis of the double-crossover mutant *S. syringae* MoS-1005. W: *S. syringae* wild type (773 bp); M: mutant strain *S. syringae* MoS-1005 (1,562 bp); Marker: DNA molecular ladder. **(C)** Metabolite profiles of *S. syringae* MoS-1005 analyzed by HPLC. 1: nocamycin I, 2: nocamycin II, 5: the newly produced derivative.

To investigate the exact role played by NcmD in nocamycin biosynthetic pathway, we firstly inactivated *NcmD* by replacing partial internal *NcmD* with *aac(3)IV* gene cassette through λ-RED recombination technology and generate *ΔNcmD* mutant strain (Figure 3A). After verifying the genotype of *S. syringae* MoS-1005 (Figure 3B), the mutant strain was cultured, and the broth was extracted for HPLC analysis. The results of HPLC revealed that three peaks with retention time of 15.1, 16.9, and 19.8 min showed UV absorption characteristics of nocamycin were detected in *S. syringae* MoS-1005 (Figure 3C). The peaks at 15.1 and 19.8 min showed the same retention time to nocamycin II and nocamycin I, respectively. Subsequently, the molecular mass of these three peaks were detected by LC-HR-MS. For the peak at 15.1 min, it had a molecular mass of 505 Dalton [*m/z* values 506.24 (M + H)^+^ and 528.2204 (M + Na)^+^; Supplementary Figure S1-A], which was identical to nocamycin II. As for the peak at 19.8 min, it had a molecular mass of 503 Dalton [*m/z* values 504.2295 (M + H)^+^ and 526.2046 (M + Na)^+^; Supplementary Figure S1-B], which was identical to nocamycin I. These results demonstrated the compounds in 15.01 and 19.8 min were nocamycin II and nocamycin I, respectively. However, for the peak at 16.9 min, it had a molecular mass of 461 Dalton [*m/z* values 462.2469 (M + H)^+^, 484.2298 (M + Na)^+^ and 945.4703 (2 M + Na)^+^; Supplementary Figure S1-C], which was different from the molecular mass of all the other nocamycin derivatives reported previously, indicating it was a new derivative produced by *S. syringae* MoS-1005.

**Figure 4:**
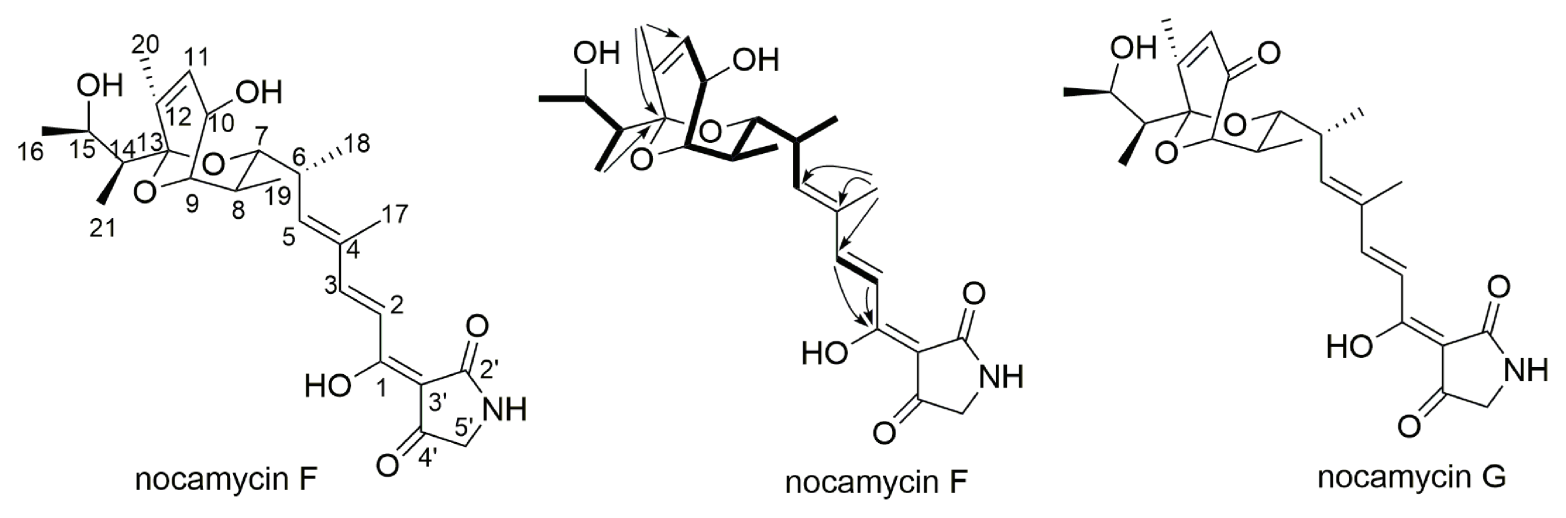
The structures of nocamycin F and nocamycin G.

To elucidate the structure of the new nocamycin analog produced by *S. syringae* MoS-1005, we purified nocamycin F from 8 L culture broth by using chemical isolation methods. The structure of nocamycin F was determined by multiple spectroscopic data analyses (Figure 4). As revealed from HR-MS (Supplementary Figure S1-C), the molecular formula of nocamycin F was determined as C_25_H_35_NO_7_, with an oxygen atom more than that of nocamycin III (Mo et al., 2017b), suggesting that nocamycin F should be a hydroxylation congener of nocamycin III. The one-dimensional NMR data exhibited marked similarities to those of nocamycin III, except that the signal of a methylene group (CH_2_-10, *δ*_C_ 23.9) in nocamycin III was absent from the NMR spectra of nocamycin F (Table 1). Instead, another oxygenated CH group (CHOH-10, *δ*_C_ 68.8/*δ*_H_ 4.59) could be observed in the spectra of nocamycin F, also as established by the COSY and HMBC data (Supplementary Figure S2).

**Table 1:** ^1^H and ^13^CNMR spectroscopic data measured in MeOD for nocamyin F.

position	*δ*_C_	*δ*_H_ (*J* in Hz)
1	185.0	
2	125.3	7.50, d (15.4)
3	147.0	7.62, d (15.4)
4	136.7	
5	141.0	5.86, m
6	35.2	2.96, m
7	79.4	3.85, m
8	37.9	2.00, m
9	74.7	3.95, t (5.9)
10	68.8	4.59, m
11	132.7	5.84, m
12	Not observed	
13	102.0	
14	45.0	1.95, m
15	69.0	4.25, m
16	20.7	1.17, d (6.3)
17	12.9	1.91, s
18	17.7	1.06, d (6.9)
19	13.1	0.98, d (7.4)
20	17.7	1.64, s
21	10.9	0.79, d (7.0)
1'		
2'	180.0	
3'	Not observed	
4'	196.6	
5'	51.7	3.66, s

### *In vitro* Characterization of NcmD in Nocamycin Biosynthetic Pathway

**Figure 5:**
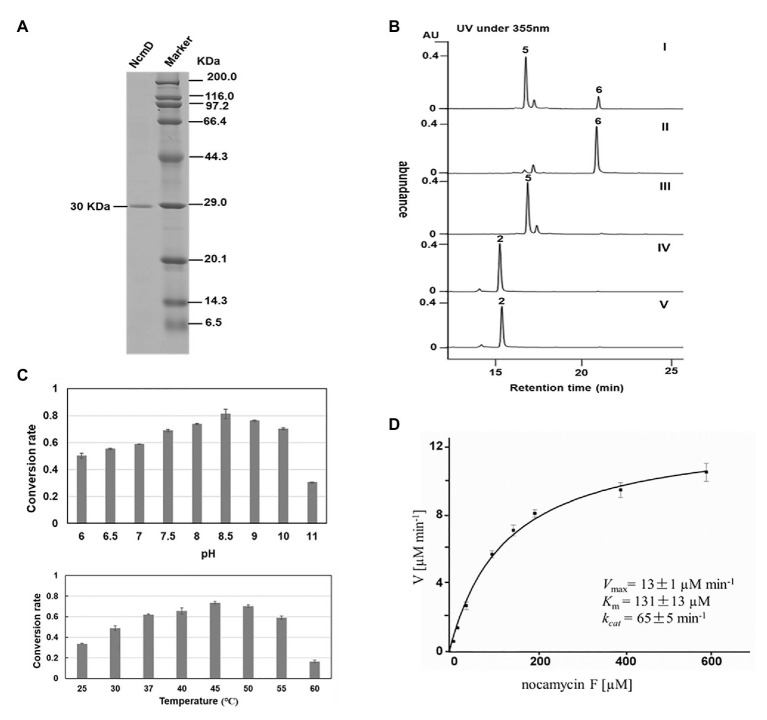
Biochemical characterization of NcmD. **(A)** Analysis of purified recombinant NcmD protein by SDS-PAGE gel. Lane 1 is the recombinant NcmD, lane 2 is protein standard. **(B)** HPLC analysis of NcmD with nocamycin *F* (5) and nocamycin II (2). I: an assay of NcmD with 5 and cofactor NADP^+^. II:an assay of NcmD with 5 and cofactor NAD^+^. III: an assay of boiled NcmD with 5 and cofactor NAD^+^. IV: an assay of NcmD with 2 and cofactor NAD^+^. V: an assay of boiled NcmD with 2 and cofactor NAD^+^. **(C)** Biochemical properties of NcmD. Upper: effects of pH on NcmD activity. Down: effects of temperature on NcmD activity. **(D)** Kinetic analysis of NcmD.

For *in vitro* characterization of NcmD activity, the protein NcmD was produced as a N-terminal His_6_-tagged protein in heterologous host *E. coli* BL21(DE3) carrying the plasmid pMoS-1005. The His_6_-tagged protein NcmD was purified to homogeneity by using Ni-NTA affinity chromatography, and SDS-PAGE analysis displayed that it had an expected molecular weight (the calculated molecular mass for NcmD is 30.078 kDa; Figure 5A). Subsequently, the catalytic properties of NcmD were investigated. At first, we detected the preference of NcmD toward cofactors NAD^+^ and NADP^+^. In the presence of NAD^+^, NcmD can efficiently catalyze substrate nocamycin F to a new compound with less polarity (Figure 5B). Whereas, in the reaction mixture with NADP^+^ instead of NAD^+^, only about 25% nocamycin F was transformed to a new compound. These results demonstrated that NcmD preferred NAD^+^ as the cofactor, which was consistent with our initial bioinformatics analyses.

To determine the structure of the new product converted from nocamycin F, we conducted a large-scale reaction of NcmD toward nocamycin F, leading to purification of the new compound 6. LC-HR-MS demonstrated that compound 6 had a molecular formula of C_25_H_33_NO_7_ with a molecular mass of 459 Dalton [*m/z* values 458.21(M-H)^−^ and 460.23(M + H)^+^; Supplementary Figure S3], two hydrogen less than that of nocamycin F. The amount of 6 obtained here was not enough for 2D NMR analysis; however, the ^13^C NMR spectra still give us sufficient structural information of 6. By comparison of ^13^C NMR data of 6 and nocamycin F, 6 showed one more carbonyl carbon (C=O, *δ*_C_ 208.8) and three oxygenated carbons (*δ*_C_ 77.8, 79.1, and 81.4), indicating that one hydrogen group in nocamycin F should be oxidized to the carbonyl group (Supplementary Figure S4). From the biosynthesis view, the difference between nocamycin F and compound 6 should be at C-10 position. For these reasons, we proposed the structure of 6 as shown in Figure 4, and it was termed as nocamycin G. Thus, NcmD was proposed to act as a dehydrogenase to catalyze the formation of ketone moiety at C-10 position.

Considering the same hydroxyl moiety harbored by nocamycin II at C-10 position, we then investigated whether NcmD can also accept nocamycin II as substrate and catalyze the conversion from nocamycin II to nocamycin I. Unfortunately, no conversion from nocamycin II to nocamycin I was observed (Figure 5B). This result demonstrated that nocamycin II was not the substrate of NcmD.

The effect of pH and temperature on NcmD properties was also investigated. For pH in the range from 6.0 to 11.0, NcmD was found to achieve maximum catalytic activity at pH 8.5, and in the range of pH 7.5–10.0, NcmD can retain activity in a high level, indicating NcmD was tolerant to pH values (Figure 5C). As for temperature, NcmD showed robust activity in the range of 37–50°C, and the optimum temperature for NcmD was 45°C (Figure 5C). Finally, we measured the steady-state kinetic parameters of NcmD toward nocamcyin F under 45°C, pH 8.5. In the presence of 2 mM NAD^+^, the *K*_m_ and *k*_cat_ values of NcmD were 131 ± 13 μM and 65 ± 5 min^−1^, respectively (Figure 5D).

## Discussion

Nocamycins belong to a small family of tetramic acid compounds bearing bicyclic ketone structure. Nocamycins demonstrate excellent antibacterial activity, especially against some anaerobic bacteria (Tsukiura et al., 1980; Tsunakawa et al., 1980; Bansal et al., 1982). Recently, we have identified the gene cluster responsible for nocamycin biosynthesis from a rare actinomycete *S. syringae* (Mo et al., 2017b). Through manipulating the gene cluster, we have generated several nocamycin analogs and characterized several gene functions involved in nocamycin biosynthetic pathway (Mo et al., 2017a,b). In this study, through *in vivo* gene inactivation and *in vitro* enzymatic assays, the SDR NcmD has been assigned to be involved in formation of ketone group at C-10 position, leading to generate nocamycin G from nocamycin F.

Short-chain dehydrogenases/reductases have been classified into seven families and the classical type is the most prominent. NcmD shows the conserved motifs belonging to the classical SDR subfamily. The majority (about 60%) of classical SDRs are expected to prefer NADP(H) (Gräff et al., 2019). For example, the SDR CAD has been shown to use NADPH as cofactor to catalyze the conversion from clavaldehyde to clavulanic acid (MacKenzie et al., 2007). The SDR Cro013448 (KP411011.1) from *Catharanthus roseus* recruits NADP(H) as cofactor in the biosynthetic pathway of plant monoterpene indole alkaloid vitrosamine (Stavrinides et al., 2018). However, the NAD^+^ dependent SDRs have also been found. FgaDH involved in ergot alkaloid fumigaclavine C biosynthetic pathway originating from *Aspergillus fumigatus* employs NAD^+^ as cofactor to catalyze the conversion from chanoclavine-I to chanoclavine-I aldehyde (Wallwey et al., 2010). Pseudoephedrine dehydrogenase (TQS88917) is a NAD^+^ dependent SDR and it catalyzes the oxidation of converted (*S*, *S*)-(+)-pseudoephedrine and (*S*, *R*)- (+)-ephedrine to (*S*)- and (*R*)-methcathinone (Shanati and Ansorge-Schumacher, 2020). For classical SDRs, the aspartic acid residue at standard position 37 has been described as a determinant of NAD(H) specificity (Belyaeva et al., 2015; Gräff et al., 2019). The current results confirm that Asp37-containing NcmD prefers NAD^+^ (Kallberg et al., 2002), which has been confirmed by resultant *in vitro* enzymatic assays. Generally, the catalytic activities decrease significantly when the SDRs are not compatible with cofactors, which have been observed from NcmD enzymatic assays, and similar results are also revealed from other SDRs (Moon et al., 2012; Takase et al., 2014; Cao et al., 2019; Gmelch et al., 2020).

**Figure 6:**
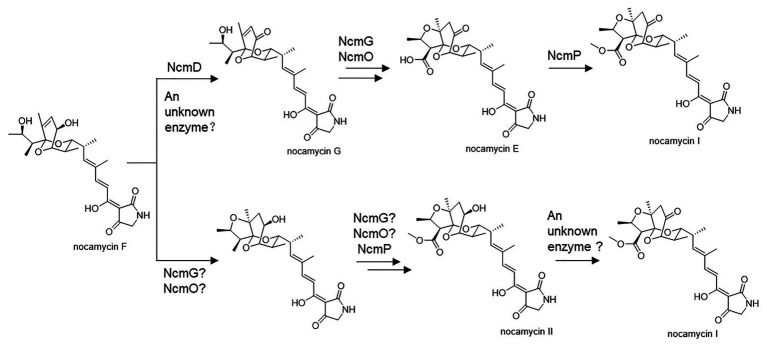
Putative post-tailoring pathway for nocamycins.

Structurally, nocamycins show high similarity to tirandamycins. Both tirandamycin B and nocamycin I harbor a ketone moiety at C-10 position. However, the proteins involved in synthesizing this moiety are significantly different. For tirandamycin B, a FAD dependent dehydrogenase TrdL catalyzes the conversion from hydroxyl moiety to ketone group at C-10 position, meanwhile, TrdL displays a flexible substrate spectrum (Mo et al., 2011). Whereas for nocamycin, the SDR NcmD shows substrate selectivity. Though *NcmD* was inactivated in the strain *S. syringae* MoS-1005, we still detected the accumulation of nocamycin I and nocamycin II, two major metabolites produced by the wild type *S. syringae*. Nocamyin II is a major metabolite in *S. syringae* wild type, which indicates the other tailoring enzymes such as NcmG, NcmO, and NcmP show flexible substrate selectivity, thus, it is rational that nocamycin II can be detected in *S. syringae* MoS-1005. For production of nocamycin I in *S. syringae* MoS1005, two possible pathways are proposed (Figure 6). Firstly, an unknown enzyme located in the genome elsewhere can compensate for the function of NcmD and catalyze the conversion from nocamycin F to nocamycin G, which then undergoes several tailoring steps to generate nocamycin I. Secondly, an unknown enzyme can catalyze the transformation from nocamycin II to nocamycin I. No matter which strategy has been employed, nocamycin I can be produced in *S. syringae* MoS1005. Taking these results together, we envision a plausible tailoring process from the formation of the intermediate nocamycin F, which is shown as Figure 6.

In summary, we generated *NcmD* deletion mutant strain *S. syringae* MoS-1005 and identified an important intermediate nocamycin F from this mutant strain. *In vitro* enzymatic assays have demonstrated that the NAD^+^ dependent SDR NcmD acts as a dehydrogenase and it is involved in formation of ketone moiety at C-10 position. However, NcmD shows substrate preference and it only displays catalytic activity toward nocamycin F. The results presented in this study provide new insights into nocamycin biosynthetic pathway.

## Data Availability Statement

The raw data supporting the conclusions of this article will be made available by the authors, without undue reservation.

## Author Contributions

XM and SY designed the experiment. XM, SY, and FD analysis the data and wrote the paper. XM and HZ performed the experiments. All authors contributed to the article and approved the submitted version.

### Conflict of Interest

The authors declare that the research was conducted in the absence of any commercial or financial relationships that could be construed as a potential conflict of interest.

## References

[ref1] Bansal M. Dhawan V. Thadepalli H. (1982). In vitro activity of Bu-2313B against anaerobic bacteria. Chemotherapy 28, 200–203. 10.1159/000238076, PMID: 6920296

[ref2] Belyaeva O. V. Chang C. Berlett M. C. Kedishvili N. Y. (2015). Evolutionary origins of retinoid active short-chain dehydrogenases/reductases of SDR16C family. Chem. Biol. Interact. 234, 135–143. 10.1016/j.cbi.2014.10.026, PMID: 25451586 PMC4414673

[ref3] Bown L. Altowairish M. S. Fyans J. K. Bignell D. R. (2016). Production of the *Streptomyces scabies* coronafacoyl phytotoxins involves a novel biosynthetic pathway with an F420-dependent oxidoreductase and a short-chain dehydrogenase/reductase. Mol. Microbiol. 101, 122–135. 10.1111/mmi.13378, PMID: 26991928

[ref4] Cao Z. Li S. Lv J. Gao H. Chen G. Awakawa T.. (2019). Biosynthesis of clinically used antibiotic fusidic acid and identification of two short-chain dehydrogenase/reductases with converse stereoselectivity. Acta Pharm. Sin. B. 9, 433–442. 10.1016/j.apsb.2018.10.007, PMID: 30972287 PMC6437595

[ref5] Carlson J. C. Li S. Gunatilleke S. S. Anzai Y. Burr D. Podust L. M.. (2011). Tirandamycin biosynthesis is mediated by co-dependent oxidative enzymes. Nat. Chem. 3, 628–633. 10.1038/nchem.1087, PMID: 21778983 PMC3154026

[ref6] Filling C. Berndt K. D. Benach J. Knapp S. Prozorovski T. Nordling E.. (2002). Critical residues for structure and catalysis in short-chain dehydrogenases/reductases. J. Biol. Chem. 277, 25677–25684. 10.1074/jbc.M202160200, PMID: 11976334

[ref7] Gauze G. Sveshnikova M. Ukholina R. Komarova G. Bazhanov V. (1977). Formation of a new antibiotic, nocamycin, by a culture of *Nocardiopsis syringae* sp. nov. Antibiotiki 22, 483–486. PMID: 883793

[ref8] Gmelch T. J. Sperl J. M. Sieber V. (2020). Molecular dynamics analysis of a rationally designed aldehyde dehydrogenase gives insights into improved activity for the non-native cofactor NAD. ACS Synth. Biol. 9, 920–929. 10.1021/acssynbio.9b00527, PMID: 32208678

[ref9] Gräff M. Buchholz P. C. F. Stockinger P. Bommarius B. Bommarius A. S. Pleiss J. (2019). The short-chain dehydrogenase/reductase engineering database (SDRED): a classification and analysis system for a highly diverse enzyme family. Proteins 87, 443–451. 10.1002/prot.25666, PMID: 30714194

[ref10] Hofmann L. Tsybovsky Y. Alexander N. S. Babino D. Leung N. Y. Montell C.. (2016). Structural insights into the *Drosophila melanogaster* retinol dehydrogenase, a member of the short-chain dehydrogenase/reductase family. Biochemistry 55, 6545–6557. 10.1021/acs.biochem.6b00907, PMID: 27809489 PMC5154250

[ref11] Kallberg Y. Oppermann U. Jörnvall H. Persson B. (2002). Short-chain dehydrogenases/reductases (SDRs). Eur. J. Biochem. 269, 4409–4417. 10.1046/j.1432-1033.2002.03130.x, PMID: 12230552

[ref12] Kavanagh K. L. Jörnvall H. Persson B. Oppermann U. (2008). Medium- and short-chain dehydrogenase/reductase gene and protein families: the SDR superfamily: functional and structural diversity within a family of metabolic and regulatory enzymes. Cell. Mol. Life Sci. 65, 3895–3906. 10.1007/s00018-008-8588-y, PMID: 19011750 PMC2792337

[ref13] Laskar A. A. Younus H. (2019). Aldehyde toxicity and metabolism: the role of aldehyde dehydrogenases in detoxification, drug resistance and carcinogenesis. Drug Metab. Rev. 51, 42–64. 10.1080/03602532.2018.1555587, PMID: 30514131

[ref14] Lukacik P. Keller B. Bunkoczi G. Kavanagh K. L. Lee W. H. Adamski J.. (2007). Structural and biochemical characterization of human orphan DHRS10 reveals a novel cytosolic enzyme with steroid dehydrogenase activity. Biochem. J. 402, 419–427. 10.1042/BJ20061319, PMID: 17067289 PMC1863559

[ref15] Luo W. Du H. J. Bonku E. M. Hou Y. L. Li L. L. Wang X. Q. (2019). An alkali-tolerant carbonyl reductase from *Bacillus subtilis* by gene mining: identification and application. Catal. Lett. 149, 2973–2983. 10.1007/s10562-019-02873-w

[ref16] MacKenzie A. K. Kershaw N. J. Hernandez H. Robinson C. V. Schofield C. J. Andersson I. (2007). Clavulanic acid dehydrogenase: structural and biochemical analysis of the final step in the biosynthesis of the beta-lactamase inhibitor clavulanic acid. Biochemistry 46, 1523–1533. 10.1021/bi061978x, PMID: 17279617

[ref17] Mattheus W. Gao L. J. Herdewijn P. Landuyt B. Verhaegen J. Masschelein J.. (2010). Isolation and purification of a new kalimantacin/batumin-related polyketide antibiotic and elucidation of its biosynthesis gene cluster. Chem. Biol. 17, 149–159. 10.1016/j.chembiol.2010.01.014, PMID: 20189105

[ref18] Mo X. Gui C. Wang Q. (2017a). Elucidation of a carboxylate O-methyltransferase NcmP in nocamycin biosynthetic biosynthetic pathway. Bioorg. Med. Chem. Lett. 27, 4431–4435. 10.1016/j.bmcl.2017.08.010, PMID: 28818448

[ref19] Mo X. Huang H. Ma J. Wang Z. Wang B. Zhang S.. (2011). Characterization of TrdL as a 10-hydroxy dehydrogenase and generation of new analogues from a tirandamycin biosynthetic pathway. Org. Lett. 13, 2212–2215. 10.1021/ol200447h, PMID: 21456513

[ref20] Mo X. Shi C. Gui C. Zhang Y. Ju J. Wang Q. (2017b). Identification of nocamycin biosynthetic gene cluster from *Saccharothrix syringae* NRRL B-16468 and generation of new nocamycin derivatives by manipulating gene cluster. Microb. Cell Fact. 16:100. 10.1186/s12934-017-0718-5, PMID: 28599654 PMC5466765

[ref21] Moon H. J. Tiwari M. K. Singh R. Kang Y. C. Lee J. K. (2012). Molecular determinants of the cofactor specificity of ribitol dehydrogenase, a short-chain dehydrogenase/reductase. Appl. Environ. Microbiol. 78, 3079–3086. 10.1128/AEM.07751-11, PMID: 22344653 PMC3346437

[ref22] Persson B. Kallberg Y. (2013). Classification and nomenclature of the superfamily of short-chain dehydrogenases/reductases (SDRs). Chem. Biol. Interact. 202, 111–115. 10.1016/j.cbi.2012.11.009, PMID: 23200746

[ref23] Savino S. Borg A. J. E. Dennig A. Pfeiffer M. de Giorgi F. Weber H.. (2019). Deciphering the enzymatic mechanism of sugar ring contraction in UDP-apiose biosynthesis. Nat. Catal. 2, 1115–1123. 10.1038/s41929-019-0382-8, PMID: 31844840 PMC6914363

[ref24] Shanati T. Ansorge-Schumacher M. B. (2020). Biodegradation of ephedrine isomers by *Arthrobacter* sp. strain TS-15: discovery of novel ephedrine and pseudoephedrine dehydrogenases. Appl. Environ. Microbiol. 86:e02487–19. 10.1128/AEM.02487-19, PMID: 31900306 PMC7054094

[ref25] Shanati T. Lockie C. Beloti L. Grogan G. Ansorge-Schumacher M. B. (2019). Two enantiocomplementary ephedrine dehydrogenases from *Arthrobacter* sp. TS-15 with broad substrate specificity. ACS Catal. 9, 6202–6211. 10.1021/acscatal.9b00621

[ref26] Sonawane P. D. Heinig U. Panda S. Gilboa N. S. Yona M. Kumar S. P.. (2018). Short-chain dehydrogenase/reductase governs steroidal specialized metabolites structural diversity and toxicity in the genus *Solanum*. Proc. Natl. Acad. Sci. USA. 115, E5419–E5428. 10.1073/pnas.1804835115, PMID: 29784829 PMC6003347

[ref27] Stavrinides A. K. Tatsis E. C. Dang T. T. Caputi L. Stevenson C. E. M. Lawson D. M.. (2018). Discovery of a short-chain dehydrogenase from *Catharanthus roseus* that produces a new monoterpene indole alkaloid. Chembiochem 19, 940–948. 10.1002/cbic.201700621, PMID: 29424954 PMC6003104

[ref28] Su B. M. Shao Z. H. Li A. P. Naeem M. Lin J. Ye L. D. (2020). Rational design of dehydrogenase/reductases based on comparative structural analysis of prereaction-state and free-state simulations for efficient asymmetric reduction of bulky aryl ketones. ACS Catal. 10, 864–876. 10.1021/acscatal.9b04778

[ref29] Takase R. Mikami B. Kawai S. Murata K. Hashimoto W. (2014). Structure-based conversion of the coenzyme requirement of a short-chain dehydrogenase/reductase involved in bacterial alginate metabolism. J. Biol. Chem. 289, 33198–33214. 10.1074/jbc.M114.585661, PMID: 25288804 PMC4246080

[ref30] Tsukiura H. Tomita K. Hanada M. Kobaru S. Tsunakawa M. Fujisawa M.. (1980). Bu-2313, a new antibiotic complex active against anaerobes I. production, isolation and properties of Bu-2313 A and B. J. Antibiot. 33, 157–165. 10.7164/antibiotics.33.157, PMID: 6900628

[ref31] Tsunakawa M. Toda S. Okita T. Hanada M. Nakagawa S. Tsukiura H.. (1980). Bu-2313, a new antibiotic complex active against anaerobes II. Structure determination of Bu-2313 a and B. J. Antibiot. 33, 166–172. 10.7164/antibiotics.33.166, PMID: 6900629

[ref32] Wallwey C. Matuschek M. Li S. M. (2010). Ergot alkaloid biosynthesis in *Aspergillus fumigatus*: conversion of chanoclavine-I to chanoclavine-I aldehyde catalyzed by a short-chain alcohol dehydrogenase FgaDH. Arch. Microbiol. 192, 127–134. 10.1007/s00203-009-0536-1, PMID: 20039019

[ref33] Zhou Y. Peng Q. Zhang L. Cheng S. Zeng L. Dong F.. (2019). Characterization of enzymes specifically producing chiral flavor compounds (R)- and (S)-1-phenylethanol from tea (*Camellia sinensis*) flowers. Food Chem. 280, 27–33. 10.1016/j.foodchem.2018.12.035, PMID: 30642496

