# Supplementary material for: Short-Chain Dehydrogenase NcmD Is Responsible for the C-10 Oxidation of Nocamycin F in Nocamycin Biosynthesis
Source: Front Microbiol. 2020 Dec 17;11:610827. doi: 10.3389/fmicb.2020.610827 (PMC7773637; doi:10.3389/fmicb.2020.610827)
Supplement: Supplementary file 1 [file Data_Sheet_1.docx]

**Supporting informations**

**Characterization of a Short Chain Dehydrogenase NcmD in Nocamycin Biosynthetic Pathway**

Xuhua Mo^1^*, Hui Zhang^1^, Fengyu Du^2^, Song Yang*^1^

^1^Shandong Province Key Laboratory of Applied Mycology, School of Life Sciences, Qingdao Agricultural University, Qingdao 266109, China.

^2^ School of Chemistry and Pharmacy, Qingdao Agricultural University, Qingdao 266109, China

*Correspondence should be addressed to X. Mo (emails: xhmo@qau.edu.cn and xhmo2013@163.com) or S. Yang (emails: yangsong1209@163.com)


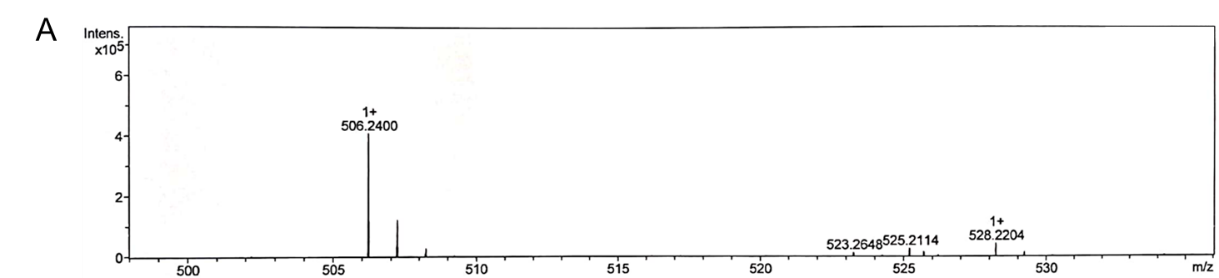


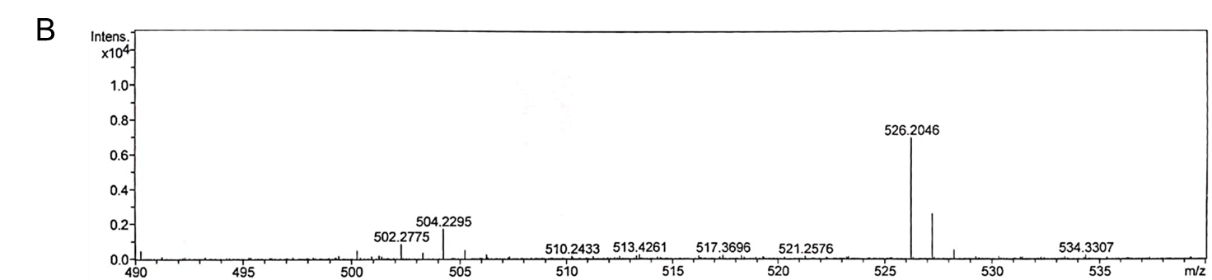


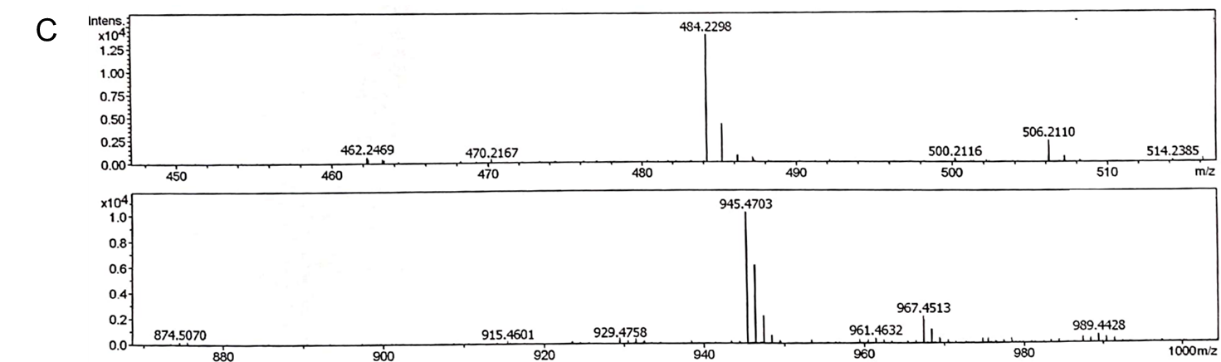


Figure S1. High resolution mass analysis of nocamycins compounds produced by *NcmD* mutant strain *S. syringae* MoS-1005. A: The mass of the peak at 15.1 min in positive pattern, m/z values 506.24 [M+H]^+^ and 528.2204 [M+Na]^+^. B: The mass of the peak at 19.8 min in positive pattern, m/z values 504.2295 [M+H]^+^ and 526.2046 [M+Na]^+^. C: The mass of the peak at 16.8 min in positive pattern, m/z values 462.2469 [M+H]^+^, 484.2298 [M+Na]^+^ and 945.4703 [2M+Na]^+^).


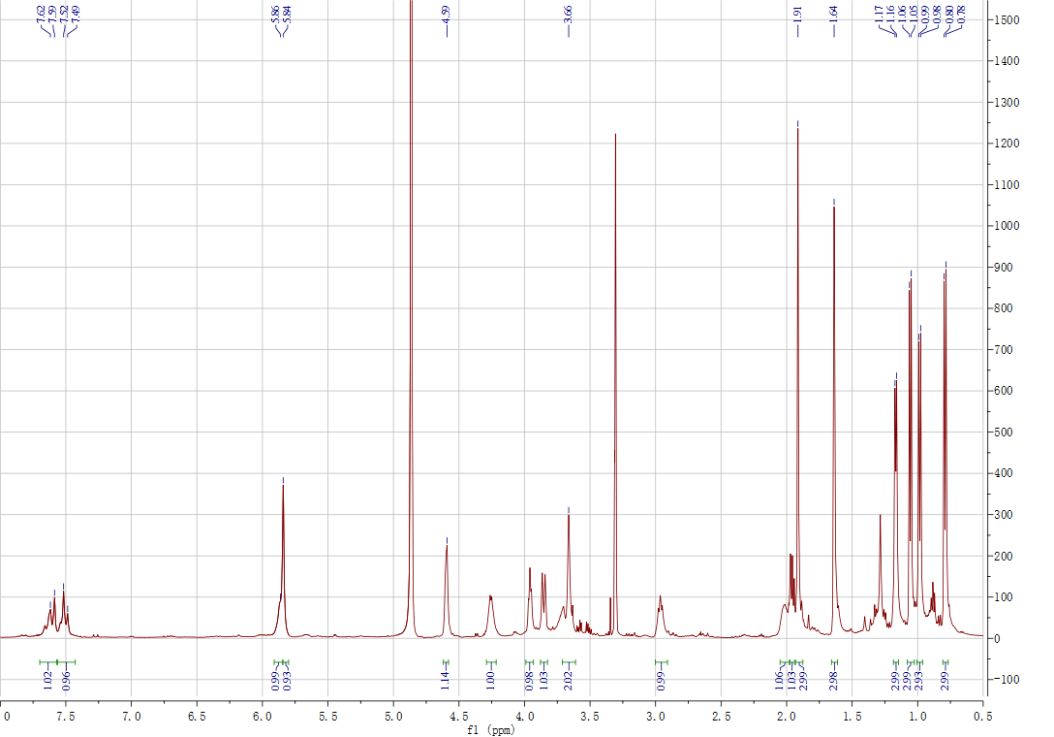


Figure S2-A. ^1^H spectrum of nocamycin F.


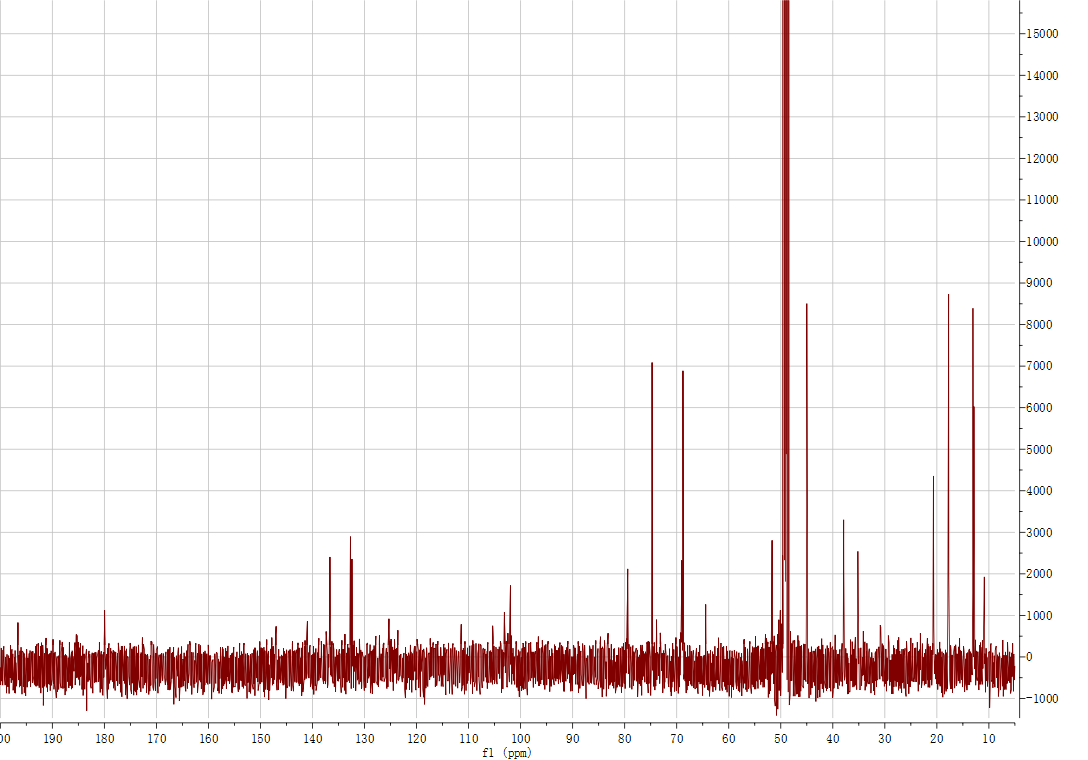


Figure S2-B. ^13^C spectrum of nocamycin F.


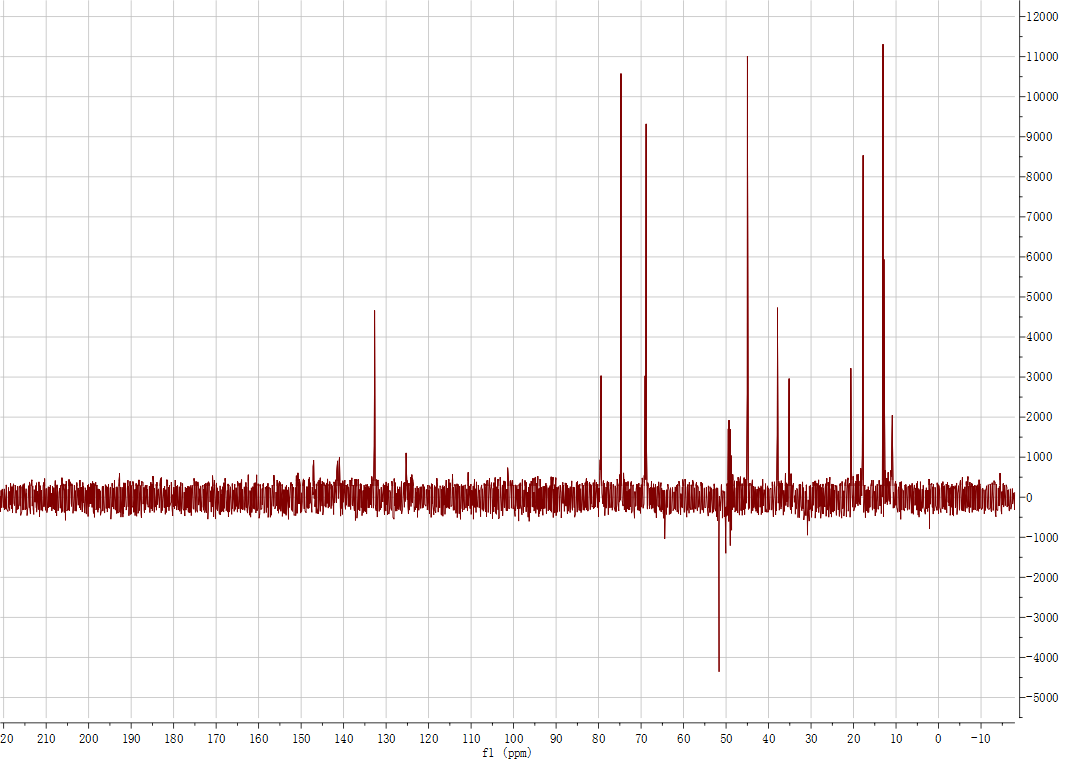


Figure S2-C. DEPT 135 spectrum of nocamycin F.


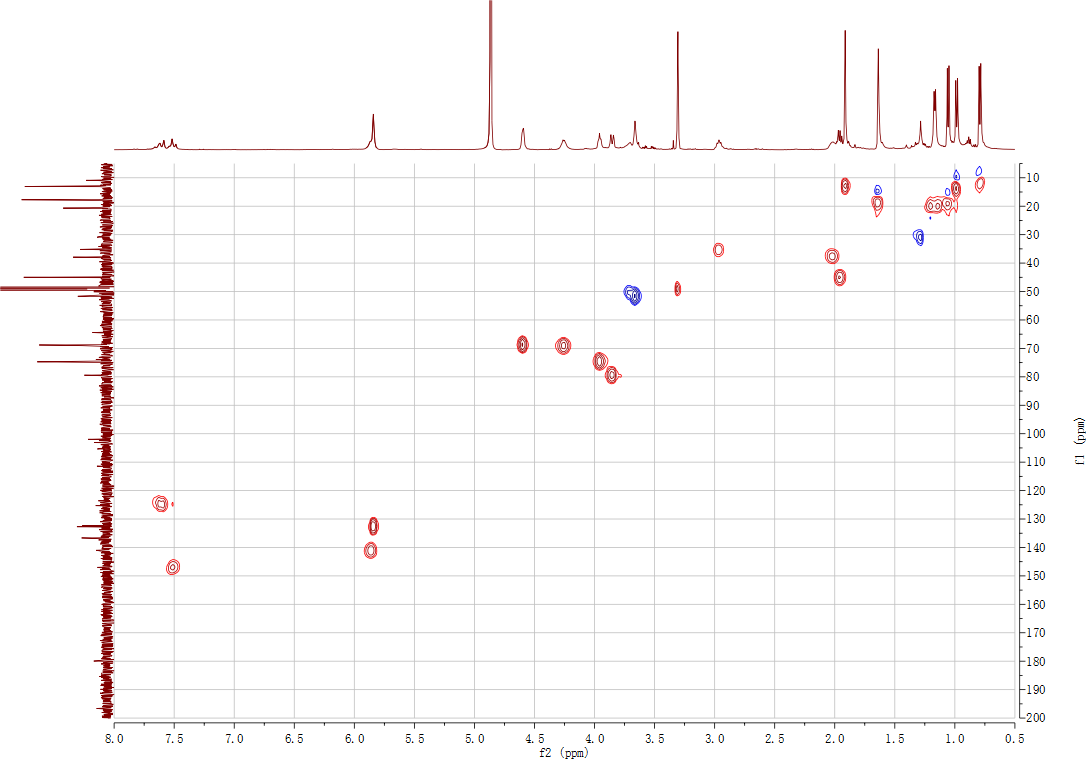


Figure S2-D. HSQC spectrum of nocamycin F.


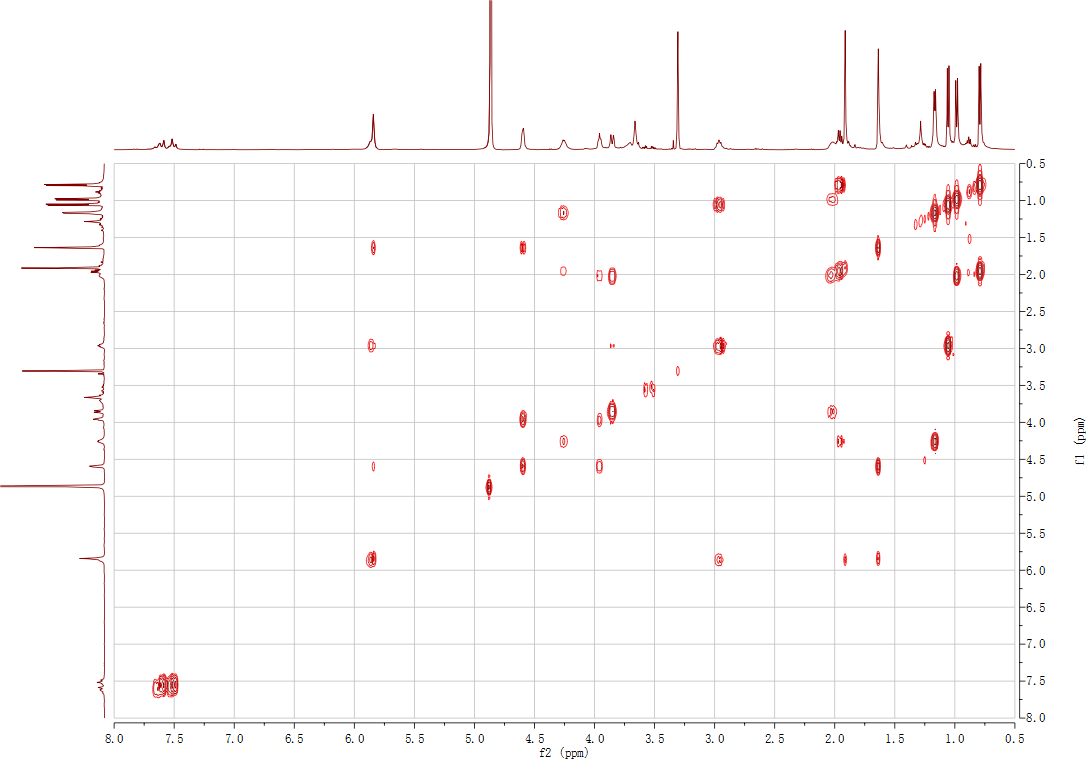


Figure S2-E. COSY spectrum of nocamycin F.


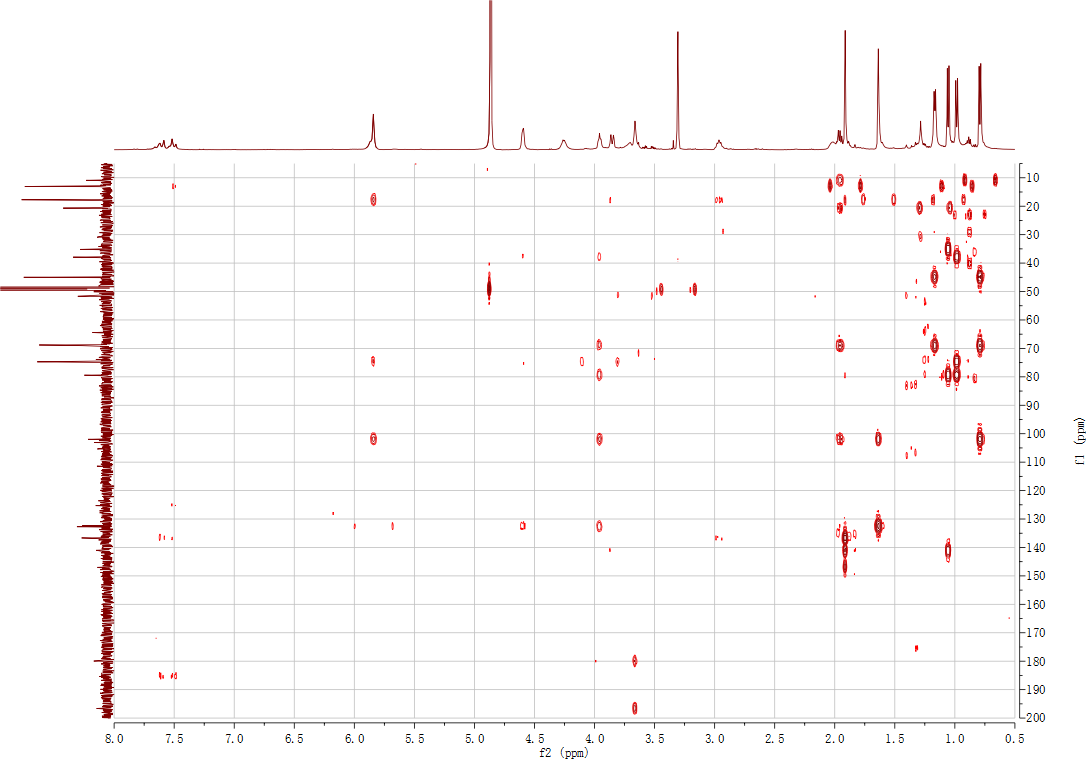


Figure S2-F. HMBC spectrum of nocamycin F.


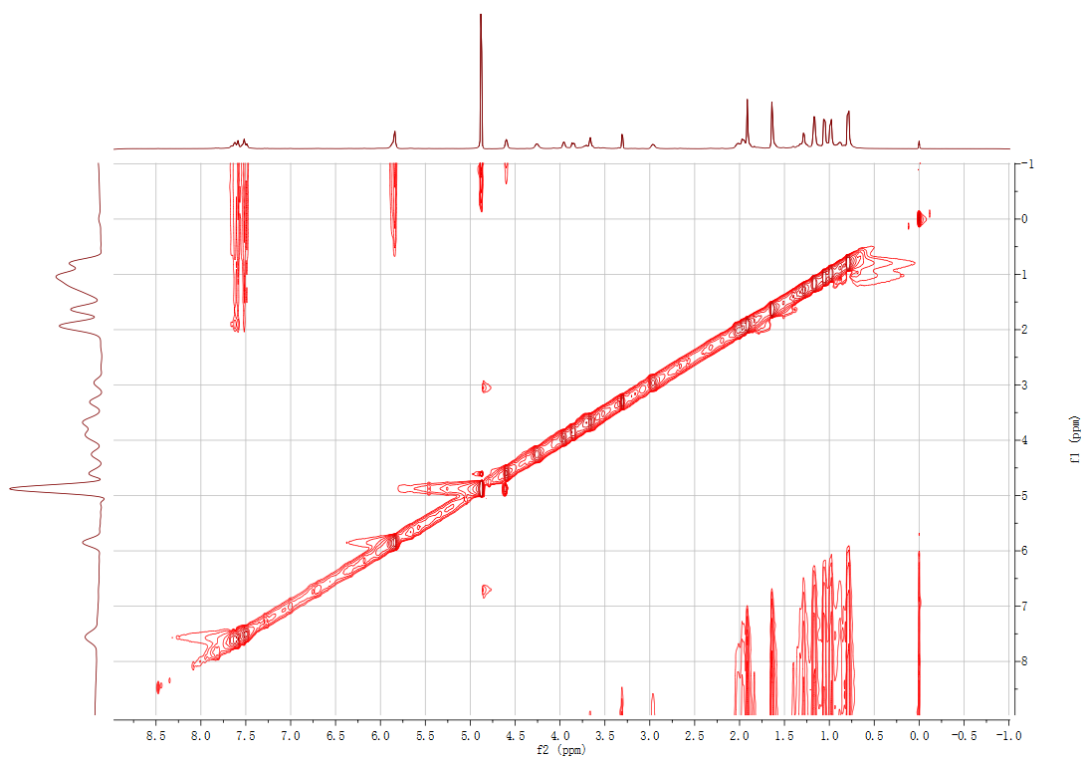


Figure S2-G. NOE spectrum of nocamycin F.


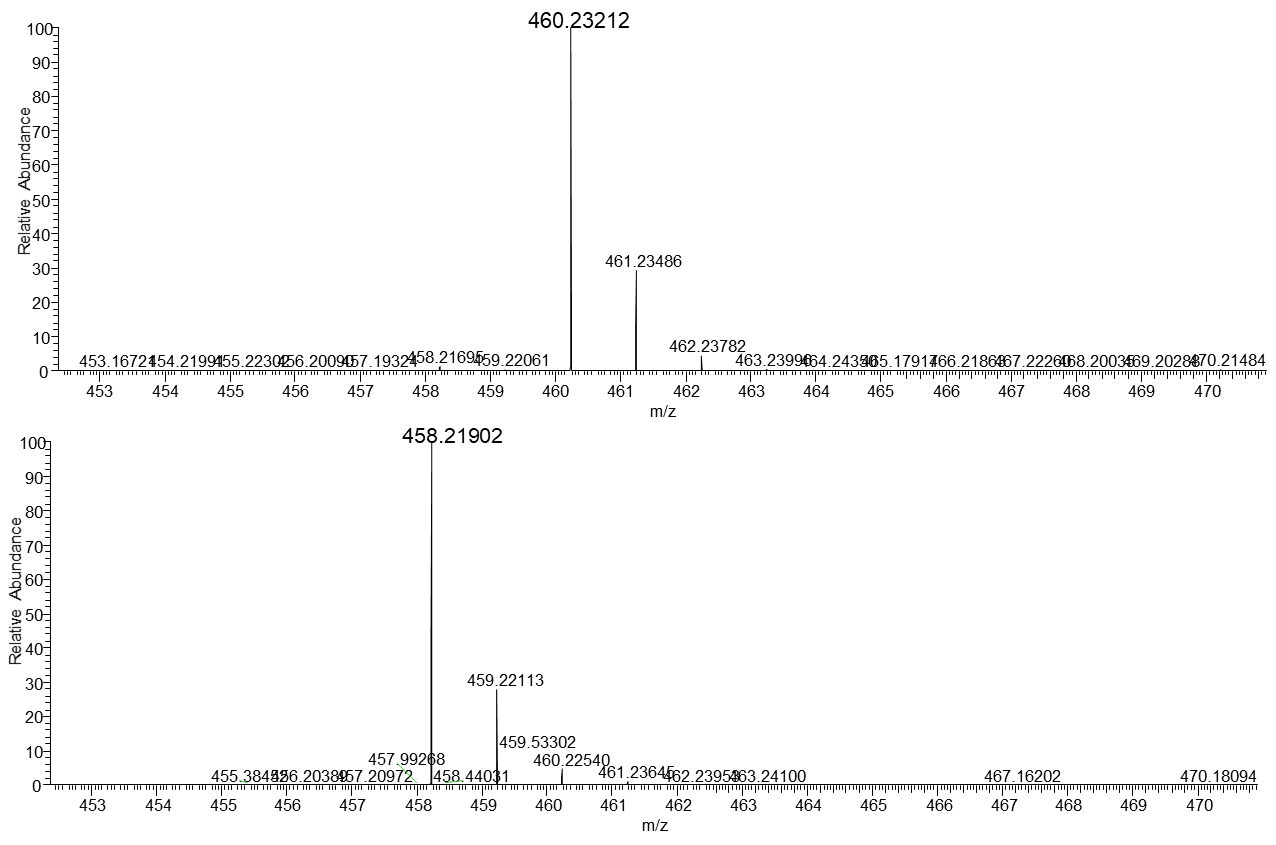


Figure S3. High resolution mass analysis of nocamycin G conversed from nocamycin F by NcmD. The m/z values 460.23212 [M+H]^+^, and m/z values 458.21902 [M-H]^-^.


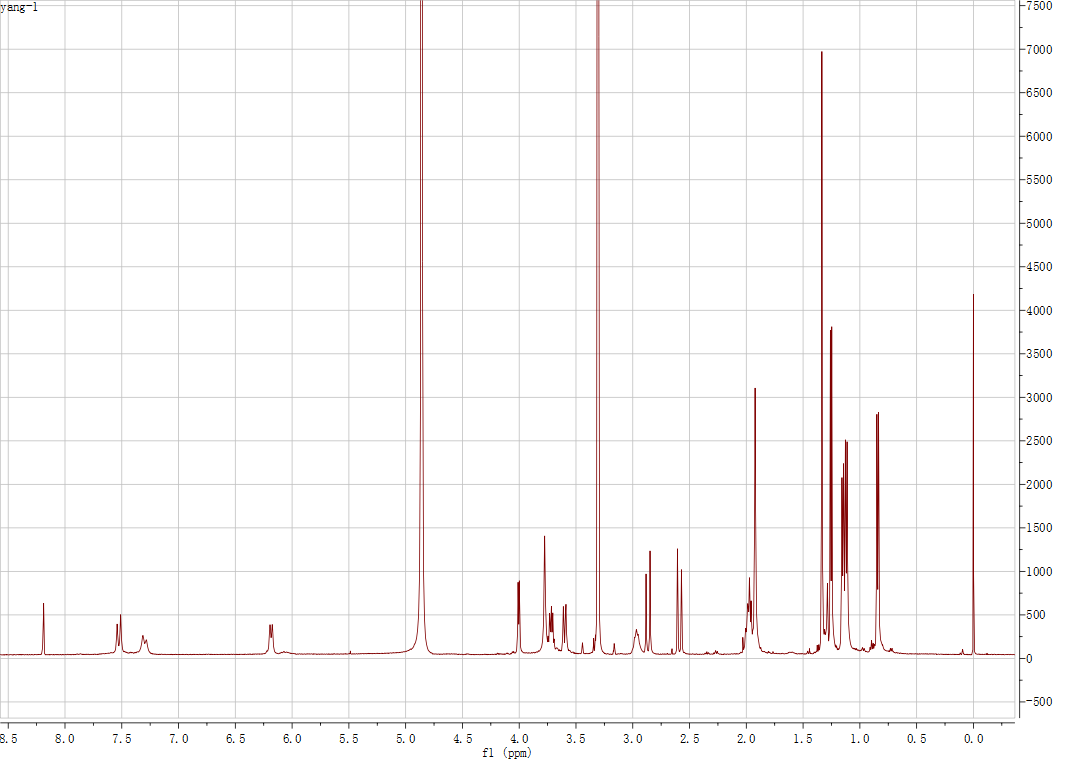


Figure S4-A. ^1^H spectrum of nocamycin G.


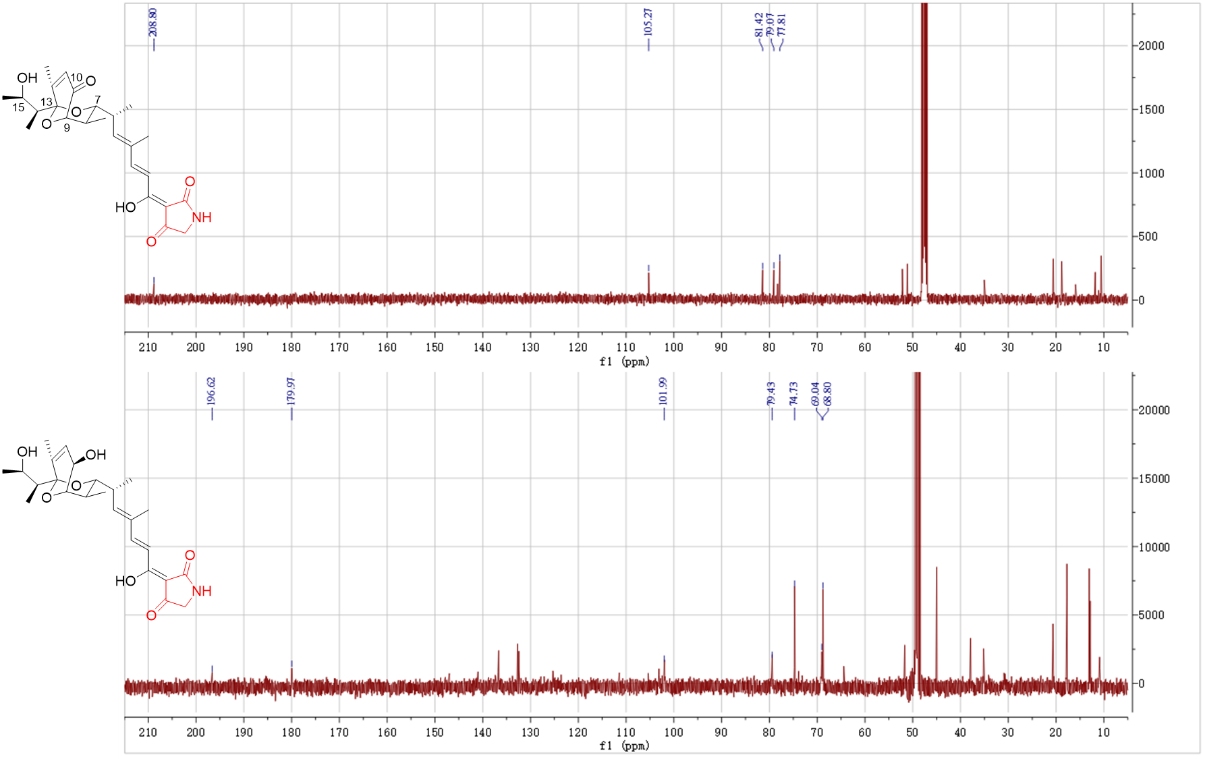


Figure S4-B. Comparison of ^13^C of nocamycin F and nocamycin G
